# Supplementary material for: A dominant-negative avirulence effector of the barley powdery mildew fungus provides mechanistic insight into barley MLA immune receptor activation
Source: J Exp Bot. 2023 Jul 20;74(18):5854–69. doi: 10.1093/jxb/erad285 (PMC10540733; doi:10.1093/jxb/erad285)
Supplement: erad285_suppl_supplementary_figures_S1-S5 [file erad285_suppl_supplementary_figures_s1-s5.pdf]

Figure S1

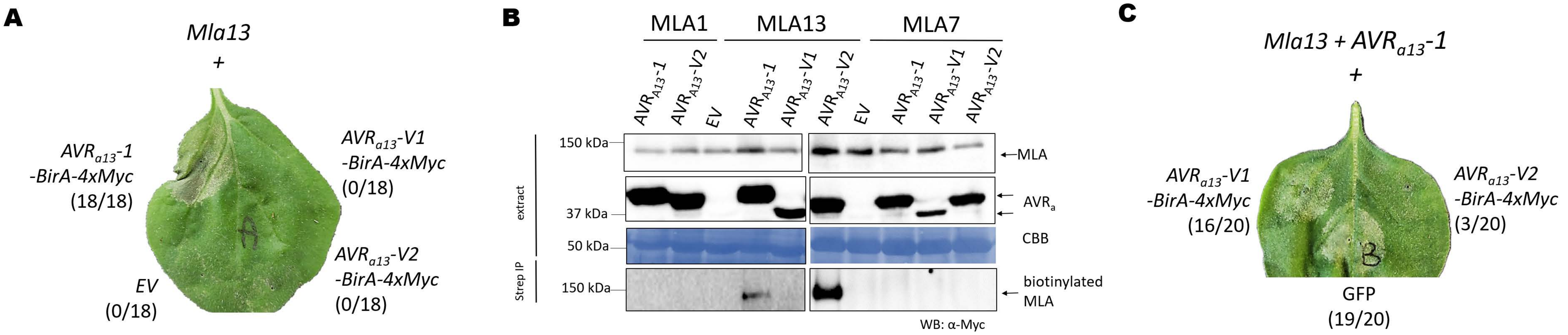

Figure S1: (A) *Nicotiana benthamiana* leaves were transformed transiently with cDNAs of the *Mla13* together with *empty vector* (*EV*) or the AVR_{a13} variants lacking SPs and fused C-terminally to *BirA-4Myc* tag sequence and expressed from the 35S promotor. Cell death was determined three days post transformation. **(B)** *N. benthamiana* leaves were transformed transiently with cDNAs of *Mla1* or *Mla7* or *MLA13* fused C-terminally to a *4xMyc* sequence and at 24 h before re-transformation with cDNAs encoding AVR_{a13}*-1-BirA-4xMyc,* AVR_{a13}*-V1-BirA-4xMyc,* AVR_{a13}*-V2-BirA-4xMyc* or *empty vector* (*EV*) as indicated. All leaves were treated with 10 µM biotin by infiltration at 24h after the second transformation. Leaf tissue was harvested 24h post biotin treatment. Total protein was extracted under denaturing conditions and recovered by Strep IP, separated by gel electrophoresis and probed by anti-Myc western blotting (WB). CBB: Coomassie brilliant blue. **(C)** *Nicotiana benthamiana* leaves were transformed transiently at a with cDNAs of the *Mla13-4Myc* and AVR_{a13}-1-BirA-4xMyc together with *GFP* or AVR_{a13}*-V1* or AVR_{a13}*–V2* lacking SPs and fused C-terminally to *BirA-4Myc* tag sequence and expressed from the 35S promotor. Agrobacteria were mixed at a 1:1:2 ratio.. Cell death was determined at three to six days post transformation.

Figure S2

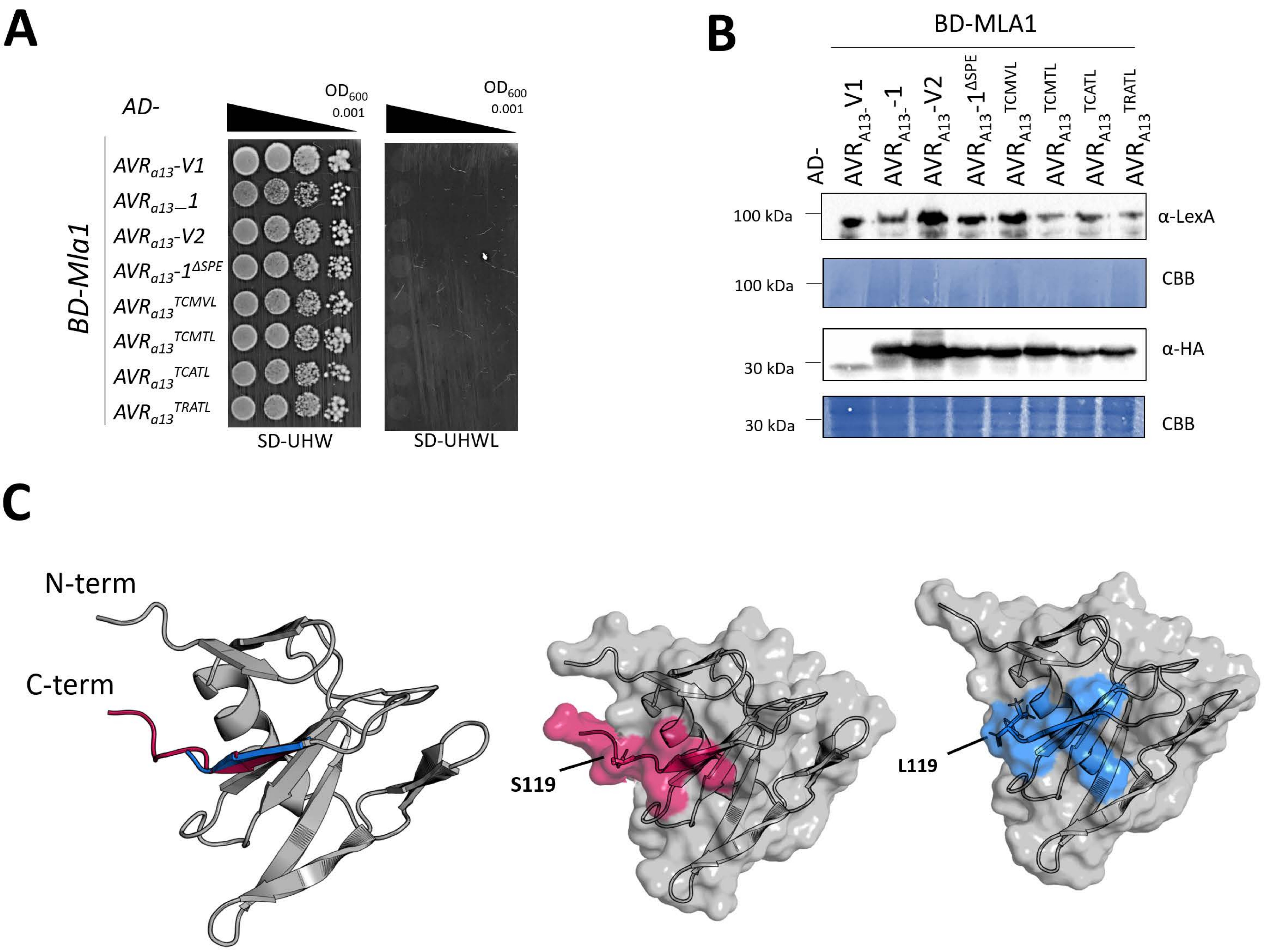

Fig. S2: (A,B) Specificity control to Figure 1D. Yeast cells were co-transformed with *Mla1* fused N-terminally to the *LexA* binding domain sequence (BD) and *AVR$_{a13}$* variants lacking SPs fused N-terminally to the *B42* activation domain (AD) and 1xHA tag sequence as indicated. Growth of transformants was determined on selective growth media containing raffinose and galactose as carbon sources but lacking uracil, histidine and tryptophan (-UHW), and interaction of proteins was determined by leucine reporter activity reflected by growth of yeast on selective media containing raffinose and galactose as carbon sources but lacking uracil, histidine, tryptophan and leucine (-UHWL). Figures shown are representatives of at least three experiments and pictures were taken 6 to 8 days after drop out. **(B)** Protein levels of BD-MLA1 and AD-AVR$_A$ variants corresponding to yeast of D. Yeast transformants were grown in raffinose and galactose containing selective media lacking uracil, tryptophan, and histidine to OD_{600} = 1. Then, cells were harvested, total protein extracted, separated by gel electrophoresis, and western blots (WB) were probed with anti-LexA or anti-HA antibodies as indicated. CBB: Coomassie brilliant blue. (C) Barley protoplasts were transfected with *pUBQ:luciferase* and genes encoding *Mla7* and either an *EV* (reference samples) or *AVR$_{a7}$-2* lacking signal peptide. Additionally, an EV or *AVR$_{a7}$-V1* lacking signal peptide were co-expressed. Luciferase activity was measured at 16 hours post transfection and relative luciferase activity determined by setting the reference samples to 1. n= 4, *p*=0.9882, n.s. = not significant. **(C)** Cartoon and surface representations for the top rank model of AVR$_{A13}$-1 and AVR$_{A13}$-V2 from AlphaFold2 (pLDDT$_{overall}$= 89, pLDDT$_{L/S119}$ >80). Residues highlighted in pink correspond to the AVR$_{A13}$-1 C-terminal residues and those in blue correspond to the AVR$_{A13}$-V2 C-terminal residues.

Figure S3

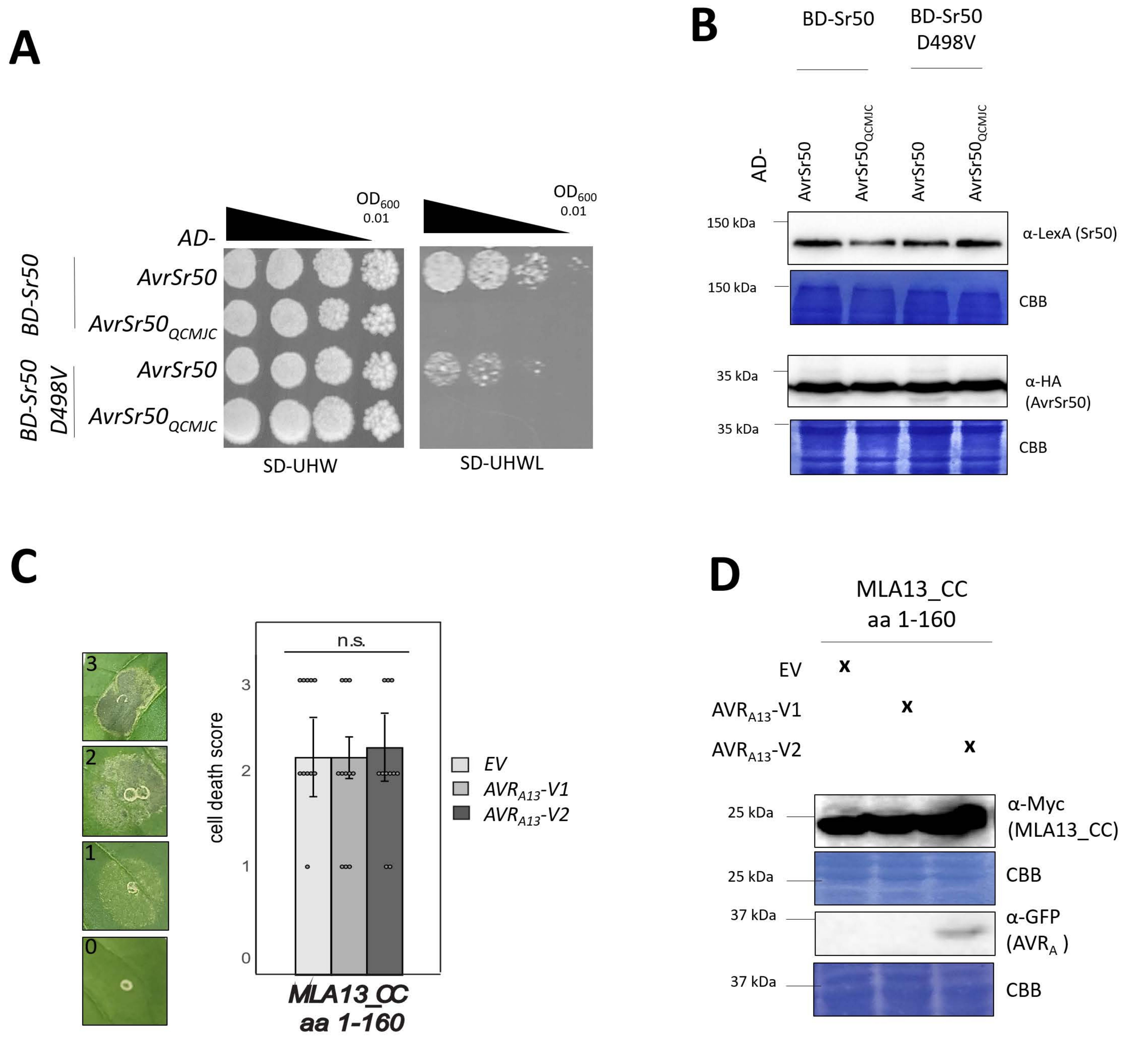

Fig. S3: (A, B) Yeast cells were co-transformed with Sr50 or Sr50 D498V fused N-terminally to the *LexA* binding domain sequence (BD) and AvrSr50 variants lacking SPs fused N-terminally to the *B42* activation domain (AD) and 1xHA tag sequence as indicated. **(A)** Growth of transformants was determined on selective growth media containing raffinose and galactose as carbon sources but lacking uracil, histidine and tryptophan (-UHW), and interaction of proteins was determined by leucine reporter activity reflected by growth of yeast on selective media containing raffinose and galactose as carbon sources but lacking uracil, histidine, tryptophan and leucine (-UHWL). Figures shown are representatives of at least three experiments and pictures were taken 12 to 14 days after drop out. **(B)** Protein levels of BD-Sr50 and AD-AvrSr50 variants corresponding to yeast of A. Yeast transformants were grown in raffinose and galactose containing selective media lacking uracil, tryptophan, and histidine to OD_{600} = 1. Then, cells were harvested, total protein extracted, separated by gel electrophoresis, and western blots (WB) were probed with anti-LexA or anti-HA antibodies as indicated. **(C, D)** *Nicotiana benthamiana* leaves were co-transformed transiently with cDNAs of *AVR$_{a13}$-V1* or *AVR$_{a13}$*-V2 or *empty vector* (*EV*) together with constructs encoding the MLA13 coiled-coil (CC) domain (aa 1-160). **(C)** Cell death was determined two days post transformation and scored from 0 to 3 based on the cell death scale indicated. All values obtained in at least three independent experiments are indicated by dots, error bars = standard error. Differences between samples were assessed by the non-parametric Kruskal-Wallis test. p= 0.623871; n.s. = not significant. **(D)** Protein levels corresponding to samples of C. Leaf tissue was harvested 36 hours post infiltration. Total protein was extracted, separated by gel electrophoresis and probed by anti-Myc (MLA13_CC) or anti-GFP (AVR$_{A13}$-V2) western blotting as indicated. CBB: Coomassie brilliant blue.

Figure S4

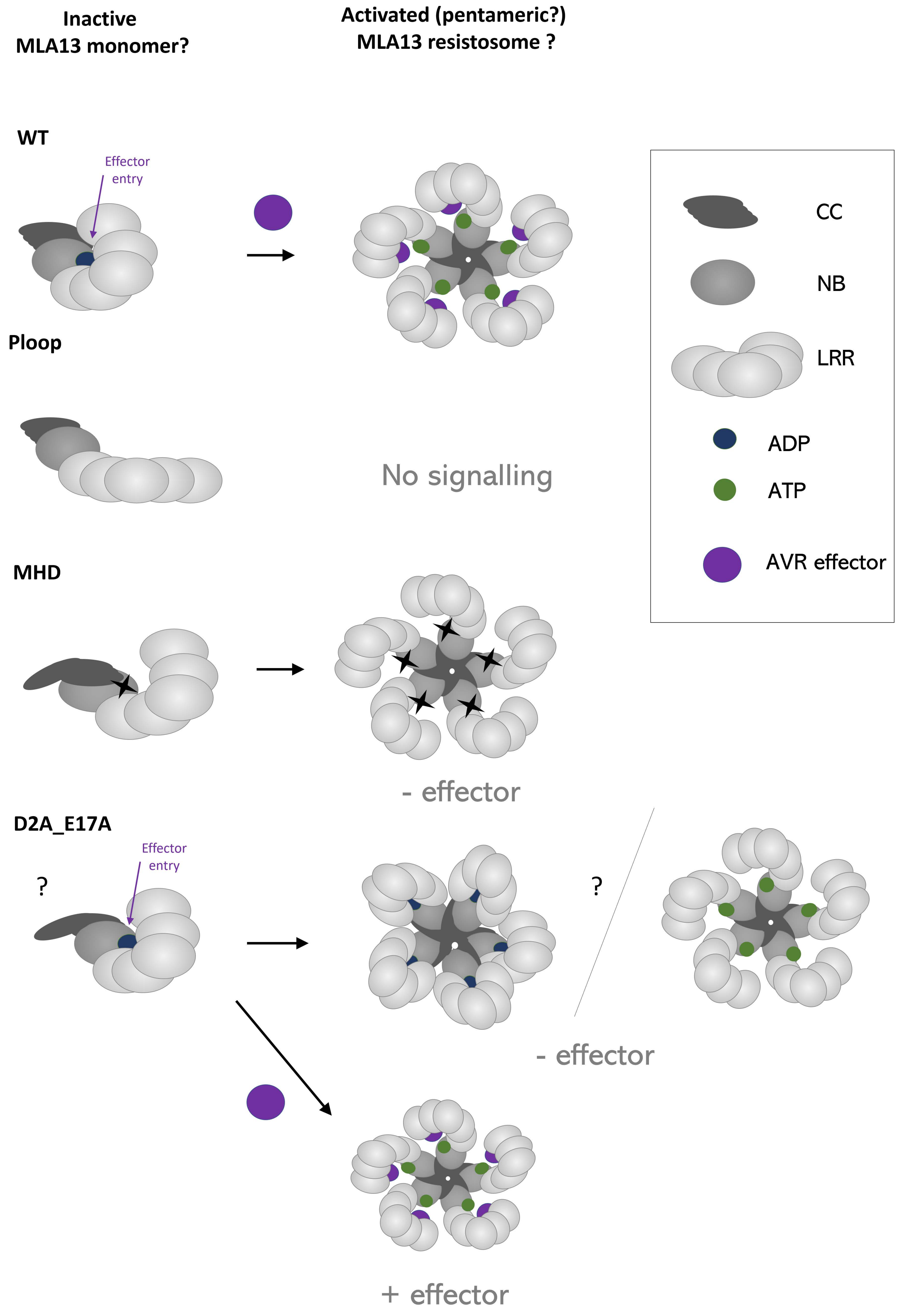

Fig. S4: Schematic hypothetical models of monomeric and oligomeric MLA13 wild type, MLA13 P-loop, MLA13 MHD and MLA13^{D2A_E17A} conformations with indication of putative effector (purple) entry sites and binding of ADP (green) or ATP (blue).

Figure S5

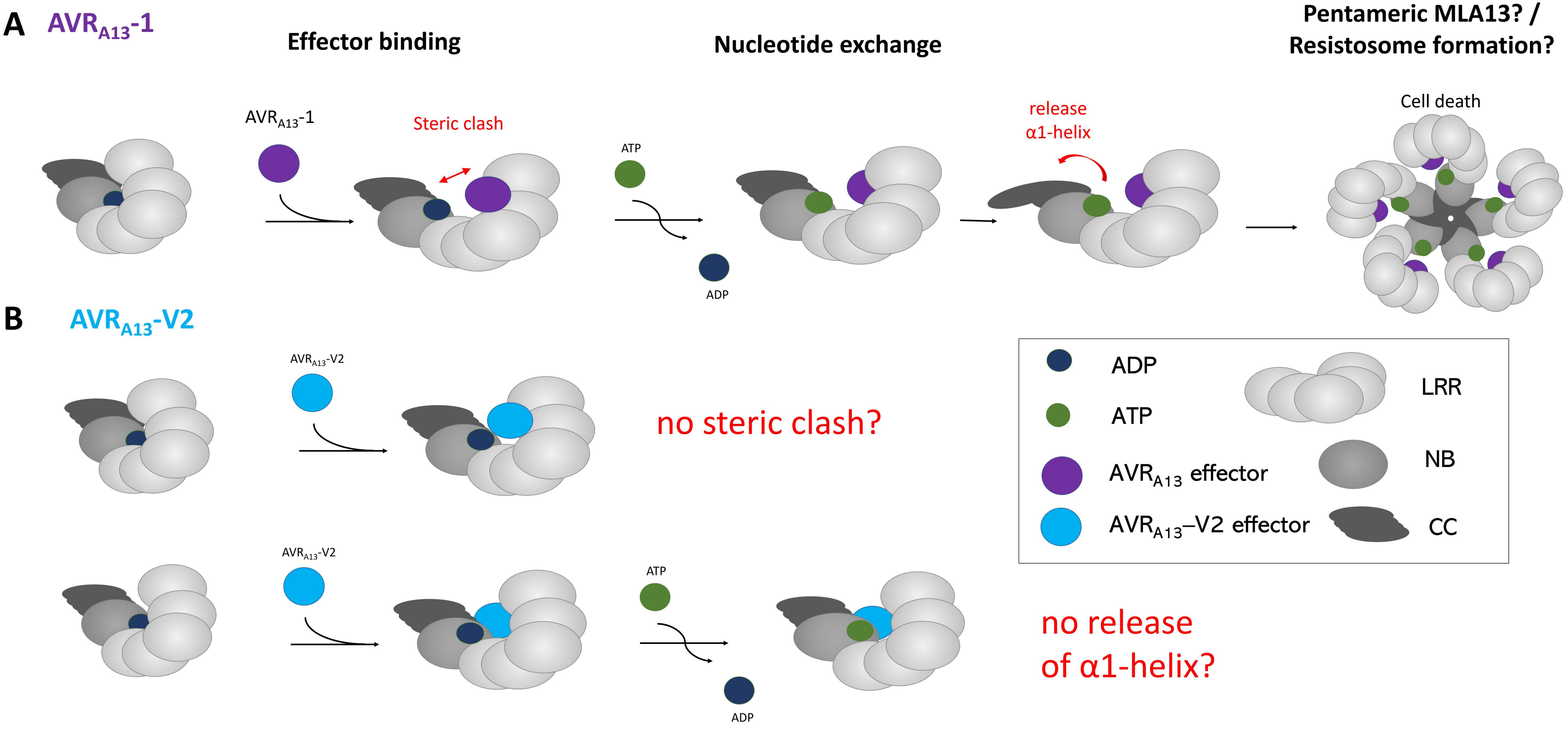

Fig. S5: Schematic models of MLA13 during the multistep process of a putative formation of a (pentameric?) resistosome initiated by the interaction with *Bgh* AVR_{A13}-1 (A) and putative models for the inhibition of the activation process by AVR_{A13}-V2 (B). (**A**) AVR_{A13}-1 binding to the effector entry point involving the MLA13 Leucine-rich-repeats (LRR) domain leads to a steric clash and subsequent replacement of adenosine diphosphate (ADP) by adenosine triphosphate ATP) in the nucleotide-binding (NB) pocket of the MLA13. ATP-binding causes additional structural rearrangement of the N-terminal Coiled-coil (CC) domain releasing the α1-helix. In the resulting putative pentameric wheel-like MLA13 resistosome, the α1-helices are thought to form a funnel like structure. (**B**) AVR_{A13}-V2 binding is likely either incapable to inducing a steric clash or prevents subsequent release of the α1-helix.
